# Diagnosis and typing of influenza using fluorescent barcoded probes

**DOI:** 10.1038/s41598-017-18333-7

**Published:** 2017-12-22

**Authors:** Bixing Huang, Nicholas P. West, Jelena Vider, Amanda J. Cox, Tanya Constantino, Bruce J. Harrower, Alyssa T. Pyke, Jamie McMahon, Judith A. Northill, Tim Riordan, David Warrilow

**Affiliations:** 1 Public Health Virology Laboratory, Queensland Health Forensic and Scientific Services, PO Box 594, Archerfield, Queensland 4108 Australia; 2 Menzies Health Institute Queensland and School of Medical Science, Griffith University, Southport, Queensland 4222 Australia; 3 NanoString Technologies, Suite 2000, 530 Fairview Avenue North, Seattle, WA 98109 USA

## Abstract

In this work, we explore a new hybridization technology using barcoded probes which has large-scale multiplexing capability. We used influenza virus to test whether the technology has application in virus diagnostics. Typing of influenza virus strains is an important aspect of global health surveillance. Standard typing procedures use serological or amplification-based assays performed sequentially. By comparison, the hybridization technology was correctly able to detect, type and subtype influenza A and B virus strains directly from clinical samples in a single reaction without prior virus isolation or amplification. Whilst currently not as sensitive as amplification-based assays, these results are a first-step towards application of this technology to the detection and typing of influenza and other viruses.

## Introduction

Both seasonal and pandemic influenza are a major problem for human health. In 2017, there were four predominant seasonal strains circulating globally; two each of influenza A and B^1^. These strains are sub-typed based on similarity to prototype strains previously identified by the World Health Organization (WHO). Influenza A strains are generally designated by the hemagglutinin (H) and neuraminidase (N) surface proteins as A-HxNy; where x and y are numbers in various combinations up to 18 and 11, respectively^2^. The current type strains for seasonal influenza A are A-H3N2 and the seasonally adapted pandemic strain A-H1N1pdm09. There are no equivalent surface protein designations for influenza B. The current circulating strains of influenza B come from either the B-Yamagata or B-Victoria lineages.

Both screening and influenza typing are important aspects of routine global surveillance. Traditionally, typing has been serology based; however, this first requires isolation of the virus. Increasingly, PCR-based methods are being used for influenza genotyping as these are generally more sensitive^3,4^. Once detected, the influenza strain can then be typed and sub-typed in a sequential process. A rapid method combining detection and typing/sub-typing would assist surveillance efforts. NanoString™ is a relatively new hybridization-based technology for the specific and sensitive detection of RNA and DNA targets^5^. NanoString methods use oligonucleotide probes which, when they are bound to their target, are recognizable by the pre-determined order of their fluorescent labels arranged sequentially such they can be read like a barcode. Probe molecules (molecular barcodes) are optically detected when bound to their respective target molecules by their specific fluorescent signatures. Hence, this technology promises specific, multiplex detection and typing of pathogens, potentially directly from clinical samples without PCR amplification or virus isolation^6^.

In this study, we test the ability of custom-designed NanoString probes to detect, type and sub-type seasonal influenza viruses. We first tested the ability of the probes to detect and sub-type influenza seasonal isolates. We then determined the approximate sensitivity of the method relative to real-time RT-PCR before demonstrating the proof-of-principle diagnostic application of the technology to detect, type and sub-type influenza directly from a subset of clinical samples. Henceforth, we refer to the assay as the influenza screening and typing assay (FluST assay).

## Results

### Typing and sub-typing of influenza isolates

**Figure 1:**
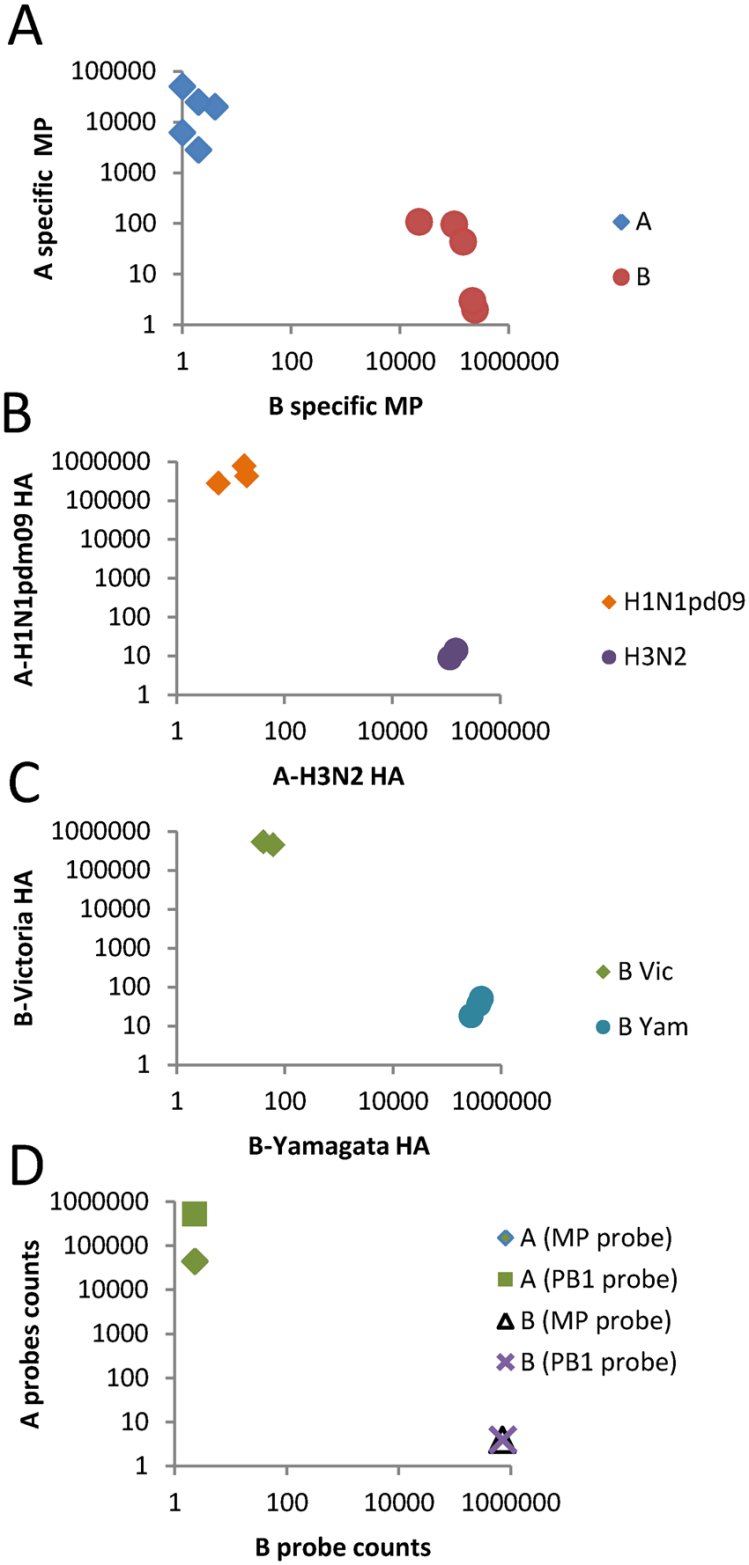
Detection, typing, and subtyping of influenza virus isolates. RNA extracts of influenza isolates were (**A**) detected and typed; and simultaneously sub-typed as strains of (**B**) influenza A or (**C**) influenza B. (**D**) A new probe based on PB1 sequence (probe 7) for the detection of influenza A is shown in comparison to the original MP probe (probe 1).

Initially, the fluorescent barcode probes 1–6 were tested on 10 influenza isolates containing influenza A: A-H1N1pdm09 (3 isolates) and A-H3N2 strains (2 isolates); and influenza B: B-Victoria (2 isolates) and B-Yamagata strains (3 isolates). RNA extracted from the isolates was hybridized to probes 1–6, washed to remove free probes, and then bound probes were counted on the nCounter machine. All probes were able to detect the respective targets to which they had been designed sufficiently to enable typing or sub-typing (Fig. 1A–C). Based on absolute counts of detected probes, the signal was highly specific, with negligible signal resulting from non-matching probes. Hence, there was clear discrimination of virus isolates by type and sub-type.

**Figure 2:**
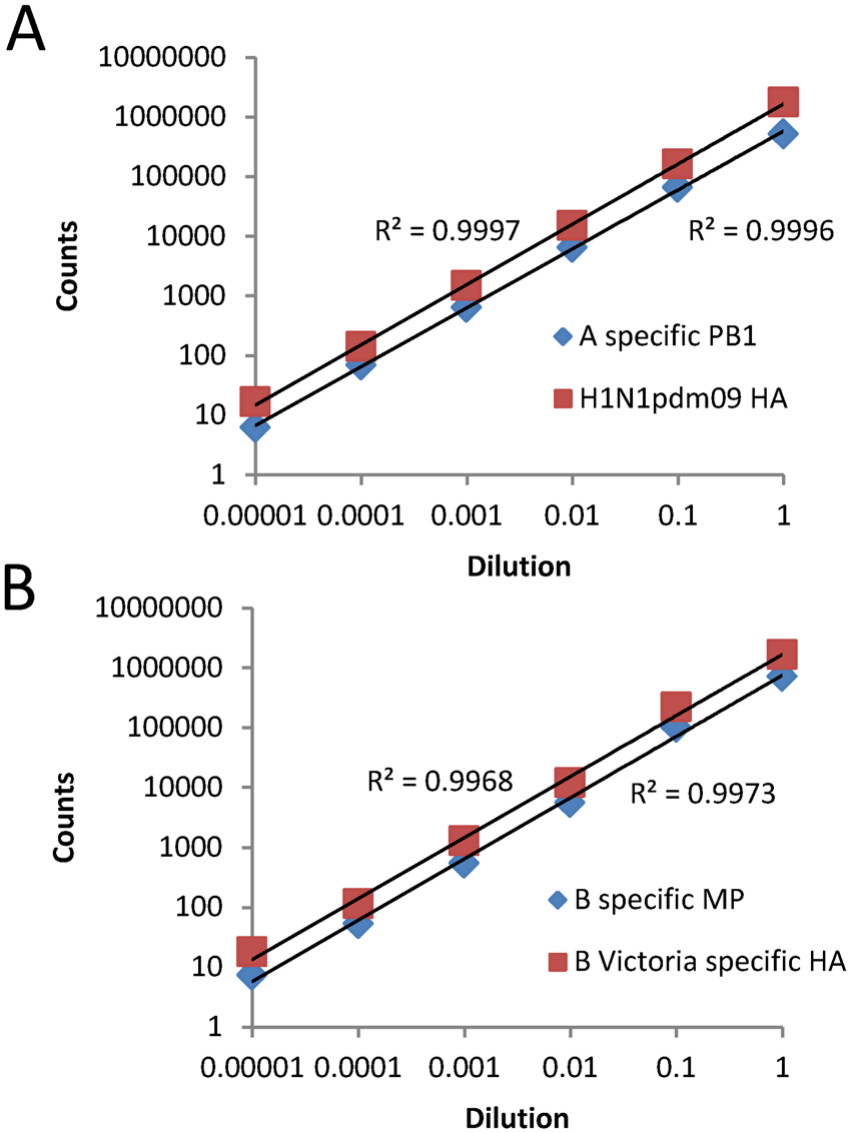
Determination of the linear range of the FluST assay. A series of six 10-fold dilutions of each of an isolate previously typed and sub-typed as either (**A**) A-H1N1pdm09 strain or (**B**) B-Victoria strain. Counts from both typing and sub-typing probes are given for each isolate.

To determine the linear dynamic range of the assay and obtain an approximate limit of detection; firstly, a 10-fold dilution series was prepared of an influenza A isolate (A-H1N1pdm09 strain) and an influenza B isolate (B-Victoria strain). The RNA extracts of these dilutions were hybridized (probes 1–7), washed and counted as previously. For this experiment, the probe mix contained the new probe to the polymerase (PB1) region of influenza A (probe 7), as well as the other probes. This probe gave notably higher counts (12 fold) than the matrix protein (MP) region probe (probe 1) and, hence, was used in subsequent experiments (Fig. 1D). In addition, counts were linear across a five-fold log_10_ dynamic range for the two strains (R^2^ ≥ 0.997) for all reactive probes (Fig. 2A,B). Counts above background were detected even for the highest dilutions of both strains (i.e. 1:10^5^ dilution). The highest dilution corresponded to detection by typing real-time RT-PCR at threshold cycle (C_T_) values of 28.1 and 29.6 for the influenza A and B strains used in this experiment, respectively.

### Fluorescent barcoded probes-based influenza virus diagnostics on clinical samples

Twenty-one respiratory samples that had been previously typed and sub-typed by standard methods were tested in the FluST assay. Influenza virus RNA was detected, typed and sub-typed in 20 of the samples (overall 95% sensitivity). The FluST assay results corresponded exactly with typing and sub-typing results obtained previously by standard methods (Table 1 and Fig. S1), and hence was completely specific. There was one sample in which influenza virus was not able to be detected or typed due to insufficient counts. This sample had the highest threshold cycle value by influenza A TaqMan typing RT-PCR (C_T_ 28). Hence, provided there is sufficient RNA in the sample (C_T_ ≤ 28), FluST is able to recapitulate the results of culture-based detection and influenza typing assays in a single reaction, without virus isolation or PCR amplification.

**Table 1:** Detection, typing and sub-typing of influenza clinical samples.

Type	Sub-type	Detection (%)	N	Ct range^*^
A	H1N1pdm09	83	6	18–28
A	H3N2	100	6	1–22
B	Victoria	100	4	19–26
B	Yamagata	100	5	18–26
Total	—	95	21	—

^*^Influenza A and B typing real-time RT-PCR.

## Discussion

Fluorescent molecular barcodes, as exemplified by the NanoString nCounter platform, can utilize complementary base pairing to specifically detect single RNA molecules of interest. As NanoString nCounter assays can be easily multiplexed, the technology has the potential for the sensitive detection, confirmation and typing of multiple pathogens and strains. For example, the technology has been used for the multiplex detection of viruses, bacteria and fungi^6^. However, the technology has not yet been applied to typing strains of a single virus such as influenza. With regard to standard typing methods, the level of multiplexing means that if multiple sequential assays can be combined into one, there may be overall time savings.

In these experiments, nCounter technology was used to develop FluST to detect and type influenza virus in clinical samples directly. In presenting these results, we are not suggesting that the FluST assay is competitive with conventional diagnostic assays in terms of sensitivity and cost. Instead, our intention was to demonstrate proof-of-principle that the technology can be applied in the setting of infectious disease diagnostics for the simultaneous detection and typing of pathogens.

The 21 clinical samples were chosen from a subset that had been previously typed and sub-typed to enable comparison between NanoString and conventional culture-based methods. Hence, the samples chosen represented a subset with higher levels of virus than are routinely found among a random sample of specimens; and influenza virus RNA was detected at an overall sensitivity of 95%. Despite the enriched subset, the FluST assay was unable to detect influenza virus in one sample, and this sample had a cycle threshold value (C_T_ 28) by typing real-time RT-PCR. This corresponded with our approximate limit-of-detection estimation by dilution of the influenza A isolate. Hence, if samples were first screened to ensure that there was sufficient target RNA for typing, then the current assay may be useful for influenza virus surveillance purposes.

As stated previously, fluorescent barcode-based probe testing offers advantages in terms of multiplexing, with the potential for up to 800 targets. In this regard, the current work is an example of minimal multiplexing. Future assay development could incorporate greater application of multiplexing to type a particular virus, or for the detection and typing of multiple viruses as part of a panel (e.g. respiratory or gastrointestinal viruses). In terms of sensitivity, a single molecule of probe bound to a target RNA is detected; hence, the technology potentially offers sensitivity approaching PCR-based assays. However, the technology currently proved to be considerably less sensitive than PCR-based assays in our study, most likely because of the benefits from amplification which increases the detectable signal. Future technical development and further improvements to methods steps such as increasing the concentration of sample RNA, optimization of hybridization conditions, and maximizing the counting step, for example, may increase the sensitivity of the nCounter method.

The application of fluorescent barcode probes applications to microbial diagnostics is at an early stage of development, hence there are some disadvantages relative to established amplification-based technologies that must be considered in addition to sensitivity. Firstly, probes detect their target by hybridization, which is currently for 24 hours with the standard protocol, followed by washing and counting. This is significantly longer than PCR-based assays. There is significant cost in terms of initial outlay for equipment, reagents and components which must be sourced from the manufacturer. However, with further technical developments to improve assay sensitivity and speed, the high multiplexing applications the technology offers could be very beneficial in terms of overall time-saving and convenience, and its cost per target may be competitive relative to standard single-target PCR-based assays.

## Materials and Methods

### Influenza clinical specimens and isolates

Clinical samples were submitted to Forensic and Scientific Services (FSS) for typing as part of routine surveillance. Twenty-one specimens, a mix of nasopharyngeal aspirates and throat swabs, were included: A-H1N1pdm09 (6 samples), A-H3N2 (6 samples), B-Victoria (4 samples) and B-Yamagata (5 samples). Isolates were obtained by inoculation and culture of Madin-Darby canine kidney (MDCK) cells. There were 10 isolates: A-H1N1pdm09 (3 isolates), A-H3N2 strains (2 isolates), B-Victoria (2 isolates) and B-Yamagata strains (3 isolates). Isolates and specimens were typed and subtyped using either PCR-based (Supplementary methods file 1) and/or hemagglutinin-inhibition assays^7^.

### Ethics approval

This work was approved by the Queensland Health Forensic and Scientific Services Human Ethics Committee in accordance with the Australian NHMRC National Statement on Ethical Conduct in Human Research 2007. The committee decided that the need for patient consent was waived for this study. All samples were submitted for routine influenza diagnostics and had been de-identified.

### Probe design

Probes were designed to recognize strain-specific 100 nucleotide regions from each target virus. Each probe was comprised of two user-synthesized, target-specific oligonucleotides which also contained complementary sequences to respective capture and reporter tag sequences supplied with the Elements™ XT TagSet system. Probes 1 and 2 were designed to the matrix region (MA) to type samples as either influenza A or B, respectively. Probes 3 and 4 were designed to the influenza A hemagglutinin (HA) region to sub-type influenza A positive samples as either A-H1N1pdm09 or A-H3N2 respectively). Probes 5 and 6 were designed to the influenza B HA region to sub-type influenza B positive samples as either B-Victoria or B-Yamagata, respectively). An additional influenza A probe to the PB1 polymerase region (probe 7) was designed to replace probe 1 after its relatively poor performance in an initial experiment.

### Fluorescent barcode probe assay

Isolates and clinical specimens were extracted using the QiaAMP viral RNA extraction kit (Qiagen). Influenza-specific probes were synthesized (Integrated DNA Technologies and Sigma-Aldrich) with adaptor sequences for the Elements™ XT TagSet system (Table S1). Probe hybridization, washing and counting was performed in accordance with established NanoString protocols (https://www.nanostring.com/support/product-support/support-documentation). Briefly, purified RNA (5 µL) was hybridized with the probe A and B master stocks at 65 °C for 24 hours. Bound target and probe was then captured and washed using the nCounter Prep Station using the automated protocol. A probe count was then obtained for each sample using the NanoString nCounter Digital Analyser.

### Data analysis

Gene expression data was analysed using the nSolver™ Analysis Software version 3.0 from NanoString Technologies (NanoString Technologies, WA, USA). Raw data was normalised by subtracting the geometric mean of six negative controls while technical variation was normalised through internal positive controls. A transcript was considered not detected if its mean count was below the geometric mean of the negative control counts.

### Data availability

All data generated or analysed during this study are included in this published article (and its Supplementary Information files).

## Electronic supplementary material

Supplementary information file
