# Supplementary material for: Diagnosis and typing of influenza using fluorescent barcoded probes
Source: Sci Rep. 2017 Dec 22;7:18092. doi: 10.1038/s41598-017-18333-7 (PMC5741751; doi:10.1038/s41598-017-18333-7)
Supplement: Supplementary file 1 — Supplementary information file [file 41598_2017_18333_MOESM1_ESM.pdf]

Supplementary information

Diagnosis and typing of influenza using fluorescent barcoded probes

Bixing Huang[1], Nicholas P. West[2], Jelena Vider[2], Amanda J. Cox[2], Tanya Constantino[1], Bruce J. Harrower[1], Alyssa T. Pyke[1], Jamie McMahon[1], Judith A. Northill[1], Tim Riordan[3] and David Warrilow[1*]

[1]Public Health Virology Laboratory, Queensland Health Forensic and Scientific Services, PO Box 594, Archerfield, Queensland 4108, Australia.

[2]Menzies Health Institute Queensland and School of Medical Science, Griffith University, Southport, Queensland 4222, Australia.

[3]NanoString Technologies, Suite 2000, 530 Fairview Avenue North, Seattle, WA 98109, USA.

[*]Corresponding author

David Warrilow

Email: David_Warrilow@health.qld.gov.au

Phone: 61-7-30962898

Fax: 61-7-30962878

Supplementary figure

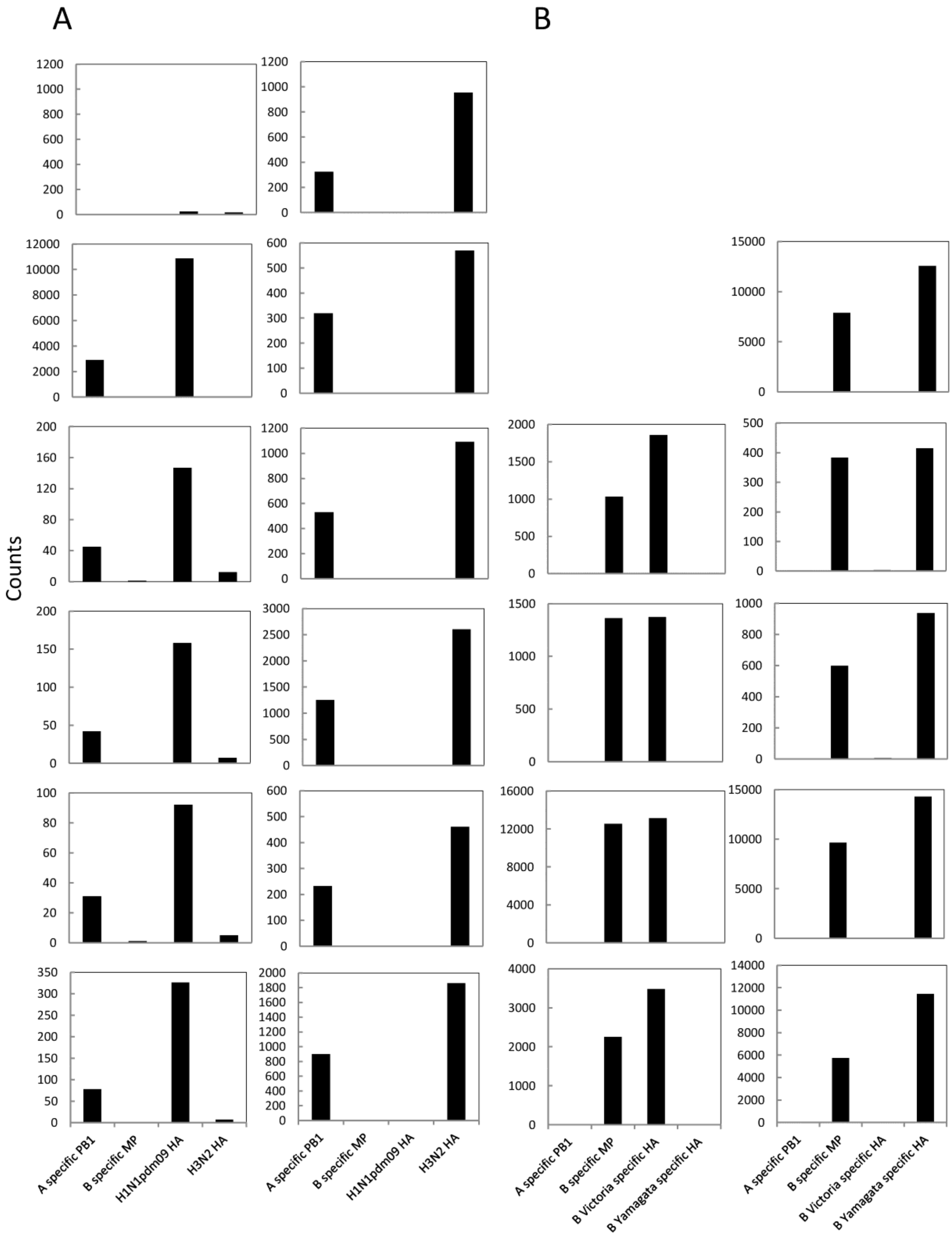

SUPPLEMENTARY FIG. 1. Typing and sub-typing of clinical respiratory samples. The FluST assay was performed on 21 respiratory samples (each graph represents a sample). The 4 columns show probe counts for (A) A-H1N1pdm09 and A-H3N2 and (B) B-Victoria and B-Yamagata, respectively.

Supplementary method

Influenza PCR-based typing and sub-typing

Briefly, 5µl of RNA extracted from patient samples using the BioRobot® Universal System and QIAamp One-For-All Nucleic Acid Kit, Qiagen, Australia) or EZ1 and Virus Mini Kit v2.0, Qiagen, Australia) were added to 15µl RT-PCR reactions (SSIII Platinum One-Step qRT-PCR System, Invitrogen, Australia; oligonucleotide sequences described in Table XX) and amplified using a RotorGene thermal cycler (Qiagen, Australia). Samples were reverse transcribed for 5 min at 50°C, incubated at 95°C for 2min then subjected to 40 cycles of 95°C for 3 sec and 60°C for 30 sec. Primer and probe oligonucleotides were synthesized by Sigma-Aldrich (Australia). Influenza B probes synthesized by Applied Biosystems (USA) Each amplification run included assay-specific primer control (5µl synthetic primer assay control RNA) and probe control (5µl synthetic probe assay control RNA) reactions constructed as outlined previously [1]. A positive result C_T was determined for each sample; a C_T value ≥40 cycles was selected to indicate no RNA was detected.

Target virus	Hybridisation orientation	Primer	Final concentration (nM)	Origin	Sequence
A/H1N1	Sense	SwFluH1fwd	900	This study	CCCCATTGCATTTGGGTAAA
	Antisense	SwFluH1rev	700	This study	TGGAGAGTGATTCACACTCTGGAT
	Probe	SwFluH1Prb	150	This study	FAM-TAACATTGCTGGCTGGATCCTGGGA-BHQ

A/H3N2	Sense	H3hFor1	500	This study	GGTACGGYTTCAGGCAT
	Antisense	H3hRev1	500	This study	TCAATCTGATGGAATTTCTCGTTG
	Probe	H3h-1144dProbe	300	[2]	FAM-CTGCTGCTTGTCCTCTTCCCT-BHQ
B/	Sense	HAB-444fw	300	[2]	ACCCTACARAMTTGGAACYTCAGG
	Antisense	*HAB-524Rv*	900	[2]	ACAGCCCAAGCCATTGTTG
	Probe	*HAB-499Probe*	250	[2]	VIC-ATCCGTTTCCATTGGTAA-MGB
	Probe	*HAB-501bProbe*	250	[2]	FAM-AAATCCGMTTTTAYTGGTAG-MGB

1. Hall-Mendelin S, Pyke AT, Moore PR, Mackay IM, McMahon JL, Ritchie SA, et al. Assessment of Local Mosquito Species Incriminates Aedes aegypti as the Potential Vector of Zika Virus in Australia. PLoS Negl Trop Dis. 2016;10(9):e0004959. doi: 10.1371/journal.pntd.0004959. PubMed PMID: 27643685; PubMed Central PMCID: PMCPMC5028067.
2. Organization WH. WHO information for molecular diagnosis of influenza virus - update: World Health Organization; 2015 [cited 2017 10/10/2017]. Available from: http://www.who.int/influenza/gisrs_laboratory/molecular_diagnosis/en/.

Supplementary table

Table S1. NanoString probes

Name	Target	Typing or sub-typing	Probe A* sequence	Probe B* sequence	Comment
**Probe 1**	A specific MP	Typing (A or B)	TTGTTTTCACGCTCACCGTGCCCAGTGAGCGAGGACTGCCTCAAGACCTAAGCGACAGCGTGACCTTGTTTCA	CGAAAGCCATGACCTCCGATCACTCGCTAAAGACAAGACCAATTCTGTCACCTTTGACTAAAGGGATTTTAGGGT	Probe B is a mix of two oligonucleotides for this target.
"	"	"	"	CGAAAGCCATGACCTCCGATCACTCGCTAAAGACAAGACCAATCTTGTCACCTCTGACTAAGGGAATTTTAGGAT	"
**Probe 2**	B specific MP	Typing (A or B)	AAAAGTTACACTGTTGGTTTGGTGGGAAAGAATTTGACCTAGACTCTGCCCATCCTCTTCTTTTCTTGGTGTTGAGAAGATGCTC	CGAAAGCCATGACCTCCGATCACTCCTACTTGCTTTCATTGACAGAAGATGGAGAAGGCAAAGCAGAACTAGCAG	
**Probe 3**	H1N1pdm09 HA	A sub-typing	CATTGCTGGCTGGATCCTGGGAAACCCAGAGTGTGAATCACTCTCCACAGCACAATTCTGCGGGTTAGCAGGAAGGTTAGGGAAC	CGAAAGCCATGACCTCCGATCACTCAAACTATGCAAACTAAGAGGGGTAGCCCCATTGCATTTGGGTAAATGTAA	
**Probe 4**	H3N2 HA	A sub-typing	TTCCAAAATGTAAACAGGATCACATACGGGGCCTGTCCCAGATATGTTAACTGTTGAGATTATTGAGCTTCATCATGACCAGAAG	CGAAAGCCATGACCTCCGATCACTCGCAAGTCTGAATGCATCACTCCAAATGGAAGCATTCCCAATGACAAACCA	
**Probe 5**	B Victoria specific HA	B sub-typing	CCAACGGAGTGACCACACATTACGTTTCACAGATTGGTGGCTTCCCGAATCCAATTTGGTTTTACTCCCCTCGATTATGCGGAGT	CGAAAGCCATGACCTCCGATCACTCAATGGCAAAGCTCTATGGGGACTCAAAGCCCCAGAAGTTCACCTCATCTG	
**Probe 6**	B Yamagata specific HA	B sub-typing	TGGAGTGGCAGTGGCGGCAGACCTTAAGAGTACACAAGAAGCTATAAATACTTTCGGGTTATATCTATCATTTACTTGACACCCT	CGAAAGCCATGACCTCCGATCACTCTGGGAAGGAATGATTGCAGGTTGGCACGGATACACATCTCACGGAGCACA	
**Probe 7**	A specific PB1	Typing (A or B)	GAGGCCATGGTGTCTAGGGCCCGGATTGATGCCAGCAACAGCCACTTTTTTTCCAAATTTTGCAAGAGCC	CGAAAGCCATGACCTCCGATCACTCTTTTCCCTAGTAGTTCATATAGGAGACCGATTGGAATTTCTAGCATGGTG	Replacement for A specific MP probe.

*probe type (not influenza A or B)
